# Improving PFAS Rejection by Ultrafiltration Membranes via Polyelectrolyte Multilayer Coating

**DOI:** 10.3390/membranes15060172

**Published:** 2025-06-07

**Authors:** Oruc Kaan Turk, Mehmet Cakmakci, Ismail Hakki Zengin, Dogan Karadag, Ebubekir Yuksel

**Affiliations:** 1 Department of Environmental Engineering, Faculty of Civil Engineering, Yıldız Technical University, İstanbul 34220, Türkiyehakki.zengin@std.yildiz.edu.tr (I.H.Z.);; 2 Department of Environmental Engineering, Faculty of Engineering, Gebze Technical University, Kocaeli 41400, Türkiye

**Keywords:** PFOA, PFOS, PFAS, membrane technology, zeta potential

## Abstract

Per- and polyfluoroalkyl substances (PFASs), used since the 1940s, are persistent and carcinogenic pollutants. Water is a major exposure route; effective removal is essential. While nanofiltration (NF) and reverse osmosis (RO) are effective but costly, ultrafiltration (UF) membranes offer advantages such as lower cost and higher flux, but their relatively large pore size makes them ineffective for PFAS compounds like perfluorooctane sulfonic acid (PFOS) and perfluorooctanoic acid (PFOA). Since PFAS removal depends on both pore size and surface properties, this study investigates the effect of polyelectrolyte multilayer coatings using poly(allylamine hydrochloride) (PAH) and poly(acrylic acid) (PAA) on the zeta potential of UF membranes. Pristine UF membranes showed limited performance (UP150: ~2% for both PFOS and PFOA; UP020: 34.4% PFOS, 24.1% PFOA), while coating significantly enhanced removal (coated UP150: 45.3% PFOS, 43.4% PFOA; coated UP020: 77.8% PFOS, 73.3% PFOA). The modified UF membranes achieved PFAS removal efficiencies significantly closer to NF membranes, though still below those of RO (e.g., BW30XLE: up to 91.0% PFOS, 88.3% PFOA; NP030: up to 81.0% PFOS, 79.3% PFOA). Findings emphasize the importance of membrane surface charge and suggest that modified UF membranes offer a promising, low-cost alternative for PFAS removal under low-pressure conditions.

## 1. Introduction

The plagues of our age, “per- and polyfluoroalkyl substances (PFASs)”, were first introduced in 1947, including compounds like perfluorooctanoic acid (PFOA), also known as “C8”. Today, over 4000 different PFAS compounds are known. This family of synthetic chemicals represents a major class of contaminants of emerging concern (CECs) [1]. Owing to their remarkable environmental permanence and resistance to degradation, they are frequently referred to as “forever chemicals” [2]. Among thousands of PFAS species, PFOA and perfluorooctane sulfonic acid (PFOS) are the most widely used. PFOS is found in numerous household and industrial products, including paints, varnishes, polishes, detergents, carpet cleaners, mobile phones, printers, and cameras, as well as in food packaging materials—raising serious concerns about food contact safety. PFOA, on the other hand, is used in pesticides, cleaning products, cable coatings, and most notably, non-stick cookware such as Teflon pans [3,4,5,6]. The primary route of exposure to these highly bioaccumulative and toxic compounds is through water sources, particularly those impacted by wastewater. As such, both wastewater and drinking water systems require effective treatment solutions to mitigate PFAS-related risks [7,8,9].

Water treatment technologies encompass a wide range of techniques, including separation and transformation processes. However, due to the strength of carbon–fluorine (C–F) bonds, PFAS compounds are highly resistant to oxidative, reductive, photolytic, microbial, and metabolic degradation, making their removal by conventional treatment methods particularly challenging [10,11,12,13]. According to the literature, the most effective treatment options for PFAS include activated carbon adsorption, ion exchange resins, and membrane-based technologies. Although activated carbon and ion exchange resins are effective, they present significant operational challenges such as limited regeneration and high replacement costs [7,13,14,15,16]. Membrane technologies, on the other hand, are known for their ability to retain PFAS, but removal of short-chain PFAS compounds remains difficult. Additionally, NF and RO require high operational pressures, which increases energy costs. While UF membranes are more cost-effective, their performance in PFAS removal is typically poor due to their relatively large pore size [17,18,19,20,21,22]. However, PFAS removal is not governed solely by pore size; membrane surface properties, particularly zeta potential, play a crucial role in the rejection of these anionic contaminants. Modifying membrane surfaces to enhance their electrostatic interactions by lowering the zeta potential can potentially improve PFAS removal efficiency [12,23]. Thus, surface modification of UF membranes presents a promising and economical strategy for enhancing PFAS rejection under low-pressure conditions.

In recent years, membrane surface modification has become a potential method for improving membrane performance, especially in applications related to the elimination of persistent organic pollutants. Modifying the surface properties of commercial membranes can enhance attributes such as hydrophilicity, fouling resistance, and surface charge [24,25,26]. Modifying the membrane’s zeta potential is particularly crucial for the efficient removal of anionic pollutants such as PFAS, which are significantly affected by electrostatic interactions [12,23]. Consequently, diverse materials have been utilized, contingent upon the particular performance objectives. Polyelectrolyte coatings, including PAH and PAA, have garnered significant interest owing to their non-toxic characteristics, straightforward application, and capacity to form multilayered structures that can modify the membrane surface charge [27,28,29,30]. These coatings provide the precise adjustment of zeta potential and foster supplementary retention mechanisms, such as electrostatic interactions, potentially improving PFAS removal beyond mere size exclusion.

The aim of this study is to investigate the potential of surface-modified UF membranes for the removal of PFOA and PFOS from water. Among various polyelectrolyte systems available for membrane surface modification, PAH and PAA were selected due to their high charge density, environmental compatibility, and ability to form stable multilayers under acidic conditions [31,32]. These polymers possess amine and carboxyl groups, respectively, which enable post-deposition crosslinking via EDAC chemistry, enhancing the durability of the coating in aqueous systems. Their strong anionic/cationic nature under pH-controlled conditions makes them particularly suitable for creating negatively charged surfaces capable of repelling PFOS and PFOA molecules through electrostatic interactions. UF membranes were coated with alternating layers of PAH and PAA to alter their surface zeta potential and enhance electrostatic interactions with negatively charged PFAS compounds. The performance of these modified membranes was evaluated under different pressure conditions and compared to commercial NF and RO membranes. The novelty of this study lies in the comprehensive investigation of how the modification of the surface zeta potential of UF membranes through LbL deposition of PAA and PAH affects the removal efficiency of PFAS. The effect of coating is systematically evaluated under a range of operating conditions, including variations in membrane material, pore size, applied pressure, and feed concentration of PFOS and PFOA. The study aims to demonstrate that zeta potential-driven mechanisms can significantly improve PFAS retention in low-pressure systems, offering a cost-effective alternative to high-pressure membrane technologies.

## 2. Materials and Methods

### 2.1. Materials, Reagents and Experimental Conditions

All experiments were conducted using a laboratory-scale dead-end filtration apparatus (HP4750, Sterlitech, Auburn, WA, USA) with an effective membrane area of 14.6 cm^2^. The system consisted of a 2.5 L external reservoir connected to a dead-end filtration cell with a capacity of approximately 300 mL. Filtration was carried out under constant pressure using inert nitrogen gas, and permeate volumes were measured using a computer-connected precision balance to calculate flux values. Experiments were conducted at a controlled temperature of 20 ± 1 °C. Ultrapure water was used in all experiments.

Two UF and one NF membrane from Microdyn-Nadir were used: UP150 (150 kDa), UP020 (20 kDa), and NP030 (0.5 kDa), along with an RO membrane from Filmtec, BW30XLE (100 Da), as summarized in Table 1.

Synthetic feed solutions were prepared by using ultrapure water, PFOA (CAS No. 335-67-1; MW: 414.07 g/mol; solubility in water: 9500 mg/L; 97% purity, from Sigma Aldrich, Darmstadt, Germany), and PFOS (CAS No. 1763-23-1; MW: 500.13 g/mol; solubility in water: 680 mg/L; 97% purity, from SynQuest Laboratories, Alachua, FL, USA). Two different PFAS concentrations were tested: (1) 500 ng/L PFOA and 250 ng/L PFOS and (2) 2500 ng/L PFOA and 1250 ng/L PFOS.

Membrane modification was performed using a layer-by-layer dip-coating technique. Water-soluble powdered PAH (CAS No. 71550-12-4), liquid PAA (CAS No. 9003-01-4), and powdered 1-ethyl-3-(3-dimethylaminopropyl)carbodiimide (EDAC, CAS No. 25952-53-8) were supplied by Alfa Aesar (Ward Hill, MA, USA). PAH and PAA solutions were prepared at 0.01 M concentrations. Membranes were sequentially immersed in each solution for 2 min, with a 30-s rinse in ultrapure water between steps to remove excess material. The selection of PAH and PAA was based on their pH-responsive charge behavior and their compatibility with carbodiimide crosslinking chemistry, which allows for stable amide bond formation between layers, improving the mechanical and chemical robustness of the coating during filtration.

**Figure 1:**
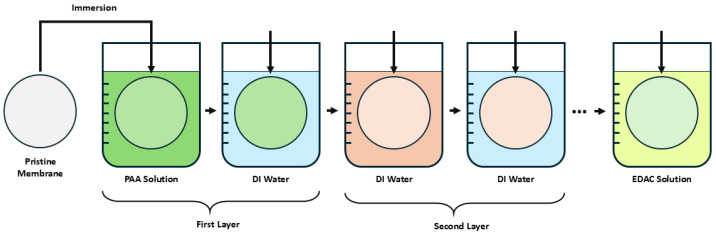
Schematic representation of the layer-by-layer dip coating process.

The interaction between PES and PAA primarily involves hydrogen bonding and electrostatic interactions, particularly under the acidic pH (3.5) conditions used during coating. Although PAA does not covalently bind to PES directly, the layer-by-layer assembly of PAH/PAA creates stable polyelectrolyte complexes. To minimize potential leaching, the final coating step includes a 0.5% EDAC treatment to induce covalent crosslinking between the carboxyl groups of PAA and the amine groups of PAH, forming stable amide bonds that enhance coating stability in aqueous environments. Regarding the impact of solution chemistry, PAA (pKa ~4.5) is mostly protonated at pH 3.5, while PAH (pKa ~8.5) remains positively charged, facilitating multilayer formation. Following the crosslinking, membranes were air-dried at room temperature for 24 h to ensure stability [12,30]. The step-by-step dip coating procedure is presented in Figure 1.

Each membrane type was operated at different transmembrane pressures (TMPs): UP020 at 1, 2, 3, and 4 bar; UP150 at 0.5, 1, 1.5, and 2 bar; NP030 at 6, 7, 8, and 9 bar; and BW30XLE at 9, 11, 13, and 15 bar.

### 2.2. Analytical Methods and Instrumentation

In all experiments, ultra-pure water was used. For PFAS analysis, the EPA Method SW-846-8327, developed by the United States Environmental Protection Agency (EPA) in 2019 and validated in 2021, was employed [33]. This method enables the analysis of 24 different PFAS compounds in surface water, groundwater, and wastewater. Analyses were conducted using a Thermo Scientific Ultimate 3000 UHPLC system coupled with a TSQ Fortis triple quadrupole mass spectrometer (LC-MS/MS from Thermo Fisher Scientific, Waltham, MA, USA). Additionally, a 5 µm, 50 × 2.1 mm PFAS delay column and a Raptor Inert C18 column (2.7 µm, 50 × 2.1 mm), both obtained from Restek (Buckinghamshire, UK), were used. Although the method allows for direct injection and rapid analysis, it also permits modifications such as solid-phase extraction (SPE) when necessary. Calibration standards and isotope-labeled surrogate standards were purchased from Wellington Laboratories. The calibration standard (PFAC-24PAR), containing 24 native PFAS compounds at 2000 ng/mL, was used to construct the calibration curve. For SPE, Resprep 28931 PFAS cartridges comprising both weak anion exchange (WAX) and carbon SPE cartridges were utilized. A 30 µL injection volume was used, and the analysis was performed using Multiple Reaction Monitoring (MRM) mode. The coefficient of determination (R²) was 0.995 for PFOS and 0.994 for PFOA. The method detection limit (LOD) was 4 ng/L, and the quantification limit (LOQ) was 1 ng/L. All samples were stored at 0–6 °C and protected from sunlight. Quality control samples such as blanks, spikes, duplicates, or recovery checks were prepared and analyzed prior to each batch.

### 2.3. Membrane Characterization

The membranes were characterized to evaluate changes in surface morphology and physicochemical properties after modification.

The application of PAH/PAA multilayer coatings was expected to modify the membrane’s molecular weight cut-off (MWCO) and porosity, thereby suggesting that PFOS/PFOA rejection could be predominantly driven by a size-exclusion mechanism. To quantitatively assess changes in membrane porosity (ε) induced by surface modification, a gravimetric approach was applied [34,35,36]. In addition, the influence of the coating on MWCO was systematically evaluated through pressure-driven filtration experiments utilizing dextran standards of well-defined molecular weights [37,38,39]. The concentrations of dextran in the feed and permeate streams were quantified using a total organic carbon (TOC) analyzer (GE Sievers M5310, from Veolia, Harrisburg, PA, USA). A series of dextran standards with different molecular weights were used to precisely determine the change in MWCO following PAH/PAA functionalization in membranes. All standards were sourced from Sigma Aldrich and used without further purification. Coating a membrane brings about changes in porosity and MWCO as well as changes in coating thickness. The film thickness depends on various factors, including chemicals used, charge density, molecular weight, temperature, depositing time, concentration, and pH of the species [40,41,42,43,44]. Although the coating thickness could not be measured during the process, to control the thickness and uniformity of the coating layer, a layer-by-layer dip-coating technique was employed using seven alternating PAH and PAA layers. The entire procedure was conducted at room temperature. These parameters were systematically optimized based on the literature to ensure consistent multilayer formation. After completion of the coating, crosslinking and drying process, the total membrane thickness was measured using a precision digital caliper to assess the physical impact of the multilayer deposition.

The analyses included scanning electron microscopy (SEM), attenuated total reflectance Fourier-transform infrared spectroscopy (ATR-FTIR), zeta potential measurements, and contact angle analysis.

SEM was performed using a Zeiss EVO^®^ LS 10 instrument (Zeiss, Oberkochen

Germany) to examine membrane surface morphology. Zeta potential measurements were carried out using a SurPASS Electrokinetic Analyzer (Anton Paar, Graz, Austria) to determine surface charge characteristics. ATR-FTIR spectra were obtained using a Perkin Elmer Spectrum 100 spectrometer (Perkin Elmer, Hopkinton, MA, USA) to identify functional groups on the membrane surface. Finally, contact angle measurements were conducted with an Attension Theta Lite tensiometer (Biolin Scientific, Stockholm, Sweden) to evaluate surface hydrophilicity.

## 3. Results

### 3.1. Effect of Coating on Membrane Surface Properities

Membrane surface chemistry plays a critical role in determining separation performance [45]. To comprehensively assess the effects of PAA/PAH surface functionalization, a series of characterization techniques were employed, including contact angle measurements, SEM, ATR-FTIR, zeta potential analysis, porosity, and MWCO determination on both pristine and used membranes.

**Figure 2:**
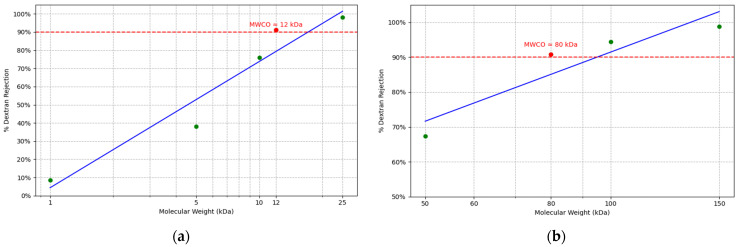
MWCO of PAH/PAA-functionalized UF membranes as determined through dextran rejections; (**a**) PAA/PAH-coated UP020 membrane; and (**b**) PAA/PAH-coated UP150 membrane.

The pristine UP150 membrane exhibited an MWCO of 150 kDa, an average contact angle of 64.95 ± 0.15°, a porosity of 69.65%, and a zeta potential of −16.8 ± 0.72 mV. Following PAA/PAH modification, the porosity decreased by 13.4% (60.31%), the MWCO was reduced by 46.7% (Figure 2), the average contact angle shifted to 54.10 ± 1.45°, and the zeta potential decreased to −32.35 ± 1.67 mV.

Similarly, the UP020 membrane initially had an MWCO of 20 kDa, an average contact angle of 69.85 ± 0.15°, a porosity of 47.25%, and a zeta potential of −13.21 ± 0.60 mV. After surface modification, the porosity decreased by 7.2% (43.85%), the MWCO was reduced by 40% (Figure 2), the contact angle decreased to 52.09 ± 1.45°, and the zeta potential was measured as −30.07 ± 1.11 mV.

The MWCO values of NP030 and BW30XLE membranes were 500 Da and 100 Da, respectively, with average contact angles of 62.80 ± 1.24° and 51.80 ± 1.98° and zeta potentials of −18.90 ± 0.88 mV and −19.40 ± 0.70 mV, respectively.

**Figure 3:**
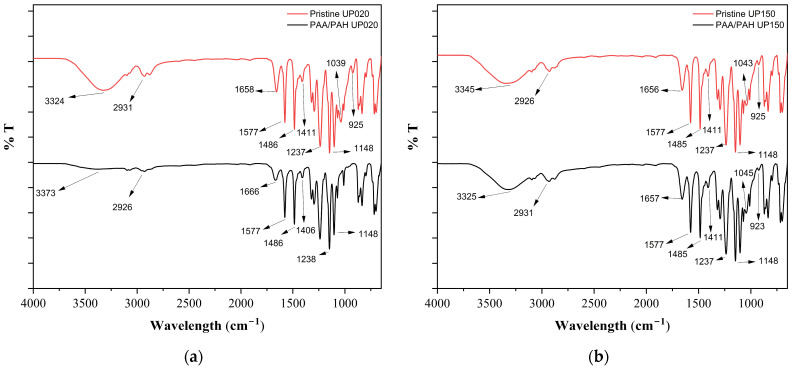
ATR-FTIR spectra: (**a**) Comparison of pristine and PAA/PAH-coated UP020 membrane; (**b**) Comparison of pristine and PAA/PAH-coated UP150 membrane.

When the ATR-FTIR spectra of pristine and PAA/PAH-coated ultrafiltration membranes are compared, the differences observed between UP020 and UP150 can likely be attributed to variations in their pore structures and surface properties (Figure 3). In the pristine UP020 membrane, characteristic PES bands at 1039 and 925 cm⁻¹ disappeared completely after coating, which may suggest that the polyelectrolyte layers formed a relatively homogeneous film on the membrane surface. In contrast, the presence of the 1043, 925, and 1237 cm⁻¹ bands in both the pristine and coated UP150 spectra implies that the chemical structure of the PES matrix remained detectable, possibly due to the limited surface coverage of the coating [46,47,48,49,50,51,52,53,54]. This observation could be associated with the larger initial pore size of UP150. Furthermore, the 46.7% reduction in MWCO observed for UP150, suggests that the coating may have penetrated further into the pores rather than just forming an outer layer. Overall, when the ATR-FTIR spectral changes and MWCO results are considered together, it can be concluded that the coating is more effective and widespread on the UP020 membrane surface, while in the case of UP150 it is probably more limited. Similar results to this study were reported by Olimattel et al., who showed that a UF membrane (UA60, ~1000 Da) functionalized with five PAH/PAA bilayers via the fluid layer-by-layer (LbL) technique showed a 38% reduction in MWCO and a 9.2% reduction in porosity. The slightly larger reductions in porosity and MWCO observed in our experiments can be attributed to the higher number of deposited bilayers and the nature of the deep coating method, which is likely to create denser and more continuous barrier layers compared to flowable LbL deposition. These findings confirm that surface modification with polyelectrolytes not only tightens membrane pores and improves size exclusion capabilities but also alters surface charge properties, which may further contribute to PFAS rejection via electrostatic repulsion [12].

Upon examining the SEM images provided in the Supplementary Material, various surface characteristics of the pristine and PAA/PAH-coated membranes were observed and analyzed. The surface of the pristine UP020 membrane does not exhibit significant roughness or large pores. In the PAA/PAH-coated UP020 membrane, however, the surface appears more homogeneous, with the coating distributed widely across the surface. The coating forms a thin film on the membrane, making the surface smoother. The pristine UP150 membrane, on the other hand, has larger surface structures and more pronounced features, with its pore structure being more distinct. In the PAA/PAH-coated UP150 membrane, however, there is a notable change on the surface, which appears smoother and more homogeneous. After coating, the distribution of particles on the surface has become more even, and irregularities have decreased. In both UP020 and UP150 membranes, it can be asserted that the PAA/PAH coating has significantly improved the surface and reduced roughness. Despite having larger pores compared to UP020, the coating effect on the UP150 membrane has also improved its surface characteristics. As a result, the coating process potentially enhances the filtration efficiency by making the surface structure of both membranes more homogeneous. Before and after the coating process, the membrane thickness was measured using a precision digital caliper. A slight but measurable increase in thickness was observed for both UP020 and UP150 membranes, confirming the successful formation of a multilayer structure. These findings, together with the observed reductions in MWCO, porosity, and contact angle, support the presence of a consistent and stable coating layer across the membrane surface.

The results demonstrate that the PAA/PAH coating rendered the membrane surface more negatively charged, as indicated by a decrease in zeta potential, and increased hydrophilicity, as evidenced by reduced contact angle values. Furthermore, porosity and MWCO analyses revealed that the more pronounced changes observed in the UP150 membrane, compared to the UP020 membrane, were attributable to its initially larger pore size. This observation is consistent with membrane characterization data.

### 3.2. Effect of Coating on PFOA and PFOS Removal

**Figure 4:**
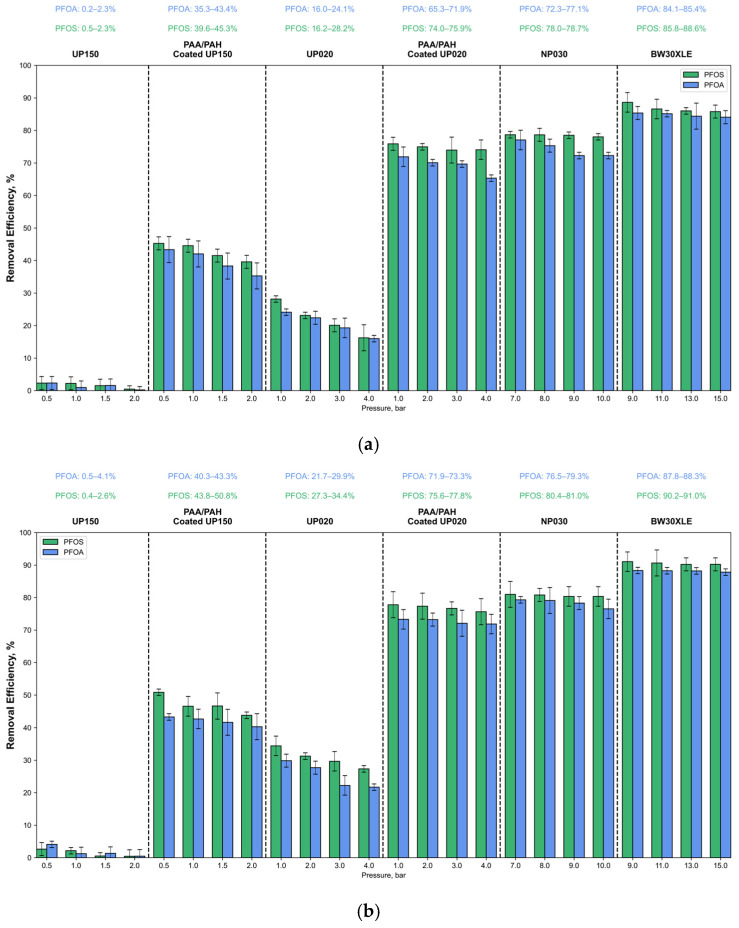
PFOA and PFOS removal efficiency: (**a**) 500 ng/L PFOA—250 ng/L PFOS; (**b**) 2500 ng/L PFOA—1250 ng/L PFOS.

Figure 4 illustrates the removal efficiencies of PFOA and PFOS across different membranes and pressures, demonstrating the significant impact of coating on enhancing removal performance for both low- and high-concentration synthetic solutions.

In the treatment of low-concentration synthetic solutions with the UP150 membrane, the removal efficiency for PFOA and PFOS ranged from 2.34 to 0.22% and 2.32 to 0.48%, respectively. Following membrane coating, the removal efficiencies for PFOA and PFOS ranged from 43.4 to 35.3% and 45.3 to 39.6%, respectively. This demonstrates that the coating increased the removal efficiency of PFOA and PFOS by approximately 35–40%. In the treatment of high-concentration solutions, the removal efficiencies for PFOA and PFOS ranged from 4.09 to 0.47% and 2.64 to 0.43%, respectively, while the removal efficiencies after membrane coating were found to be 43.3–40.3% for PFOA and 50.8–43.8% for PFOS. A slight decrease in removal efficiency was observed with increasing pressure, while a slight improvement in removal efficiency was seen with the increase in contaminant concentration.

For the UP020 membrane, in the treatment of low-concentration synthetic solutions, the removal efficiency for PFOA and PFOS ranged from 24.1 to 16% and 28.2 to 16.2%, respectively. In the treatment of high-concentration solutions, the removal efficiency for PFOA and PFOS ranged from 29.9 to 21.7% and 34.4 to 27.3%, respectively. After membrane coating, the removal efficiencies for low-concentration synthetic solutions were 71.9–65.3% for PFOA and 75.9–74.1% for PFOS, while for high-concentration solutions, the removal efficiencies were 73.3–71.9% for PFOA and 77.8–75.6% for PFOS. After coating, the removal efficiencies of both PFOS and PFOA showed a more noticeable improvement for UP020 compared to UP150. The NP030 membrane exhibited removal efficiencies for low-concentration synthetic solutions of 77.1–72.3% for PFOA and 78.7–78% for PFOS, while for high-concentration solutions, the removal efficiencies were 79.3–76.5% for PFOA and 81–80.4% for PFOS. Additionally, as expected, the BW30XLE membrane showed the highest removal efficiencies for low-concentration synthetic solutions of 85.4–84.1% for PFOA and 88.6–85.8% for PFOS, while for high-concentration solutions, the removal efficiencies were 88.3–87.8% for PFOA and 91–90.2% for PFOS. A slight decrease in removal efficiency was observed for all membranes with increasing pressure, but this decrease was lessened with the coating. This may be due to the tightening of pore size or a reduction in the rough surface structure of the membrane.

In all experiments, PFOS consistently showed higher retention than PFOA, which can be attributed to two main factors: (i) PFOS has a larger molecular size and a more delocalized charge distribution, which leads to stronger electrostatic interactions with the membrane surface, and (ii) PFOS, compared to PFOA, has a higher hydrophobicity, supporting stronger hydrophobic interactions with the membrane material [20,55,56]. Notably, the coated UP020 membrane, which exhibited a 7.2% reduction in porosity and a 40% reduction in MWCO, achieved PFOA and PFOS rejection performance comparable to that of the NP020 membrane. These findings indicate that the separation performance is more strongly influenced by surface charge (zeta potential) than by MWCO alone. In experiments conducted with synthetic wastewater containing higher concentrations of PFOA and PFOS, a slight but consistent improvement in removal efficiency was observed. This improvement can be attributed to the increased interactions on the membrane surface due to the higher pollutant concentrations. The findings are consistent with previously reported results in the literature. For instance, Wang et al. demonstrated that negatively charged poly(piperazine-amide) nanofiltration membranes achieved over 88% steady-state rejection of PFOS, which was potentially attributed to the intrinsic molecular structure of polyamide. Moreover, they reported an increase in PFOS removal efficiency with rising pollutant concentrations [57]. Similarly, Zhao et al. highlighted that a more positive membrane zeta potential enhances PFOS removal through increased adsorption, whereas a more negative surface charge improves rejection via intensified electrostatic repulsion and size exclusion mechanisms [58]. Yamamura et al. also reported PFOA and PFOS removal efficiencies exceeding 85% using RO membranes, emphasizing the dominant role of electrostatic repulsion and size-based exclusion in the separation process [59]. Tang et al., however, observed effective PFAS rejection with dense RO membranes even in the absence of significant zeta potential effects, suggesting that membrane tightness alone may be sufficient for PFAS removal [60]. In another study, Olimattel achieved approximately a 30% improvement in PFOS and PFOA rejection by coating UF membranes with five bilayers using the layer-by-layer (LbL) method, compared to unmodified membranes [12]. Likewise, Zeng found that a UF membrane with a molecular weight cut-off of 10 kDa and 27 kDa and a more negative zeta potential exhibited higher rejection tendencies for PFHxA, achieving up to 95% removal efficiency [23].

### 3.3. Membrane Autospy

A comprehensive understanding of membrane operation and potential fouling mechanisms requires the use of various characterization techniques [61,62]. SEM, ATR-FTIR, and flux measurements provide critical insights into membrane performance and structural integrity.

**Figure 5:**
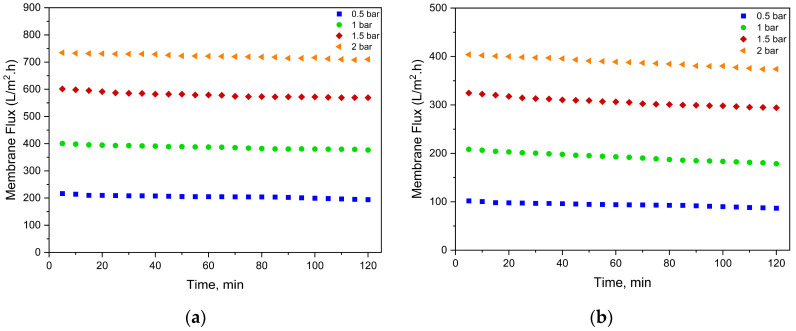

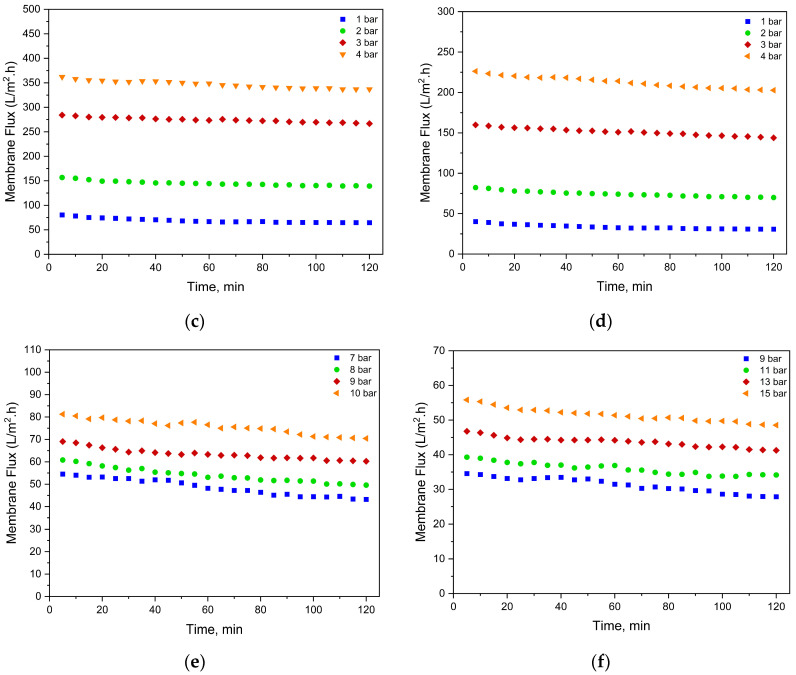
Membrane fluxes in the treatment of synthetic solution containing 500 ng/L PFOA and 250 ng/L PFOS. (**a**) UP150; (**b**) PAA/PAH-coated UP150; (**c**) UP020; (**d**) PAA/PAH-coated UP020; (**e**) NP030; (**f**) BW30XLE.

**Figure 6:**
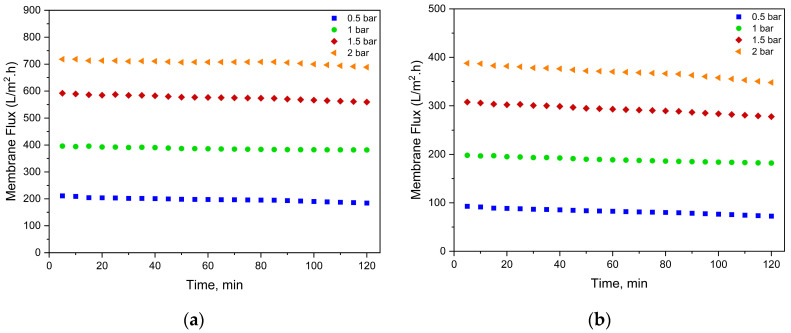

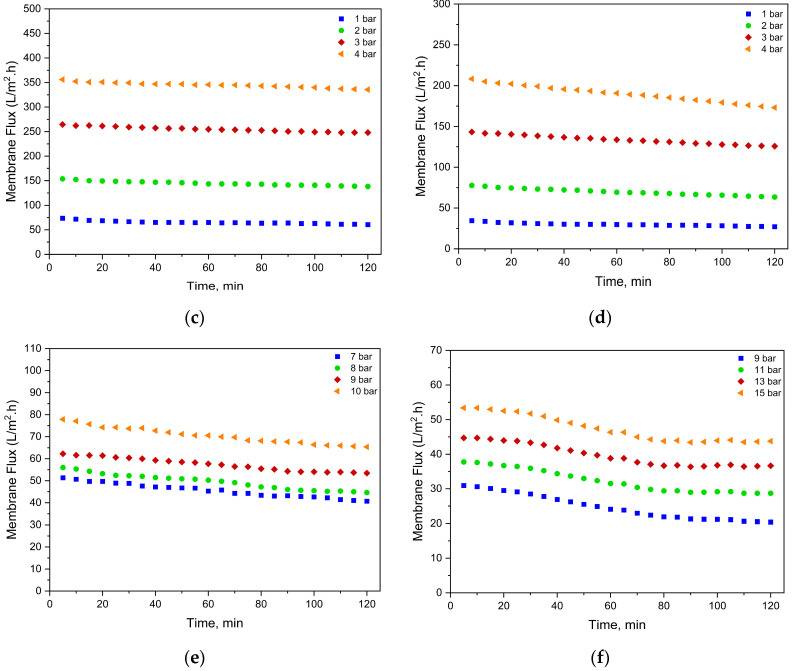
Membrane fluxes in the treatment of synthetic solution containing 2500 ng/L PFOA and 1250 ng/L PFOS. (**a**) UP150; (**b**) PAA/PAH-coated UP150; (**c**) UP020; (**d**) PAA/PAH-coated UP020; (**e**) NP030; (**f**) BW30XLE.

An important advantage of membranes with larger pore sizes compared to those with smaller pores is the higher mass transfer coefficient, leading to a significant increase in membrane flux. The results of the membrane fluxes shown in Figure 5 and Figure 6 are that the flux values increase proportionally with pressure and membrane pore size. Also, higher concentrations of PFOA and PFOS cause a decrease in the average flux. The flux remained relatively constant over the 2 h operating time for all membranes tested at each pressure.

As shown in Figure 5 and Figure 6, the average flux of the UP150 membrane during the treatment of low-concentration synthetic solution was measured as 204.84, 386.94, 579.75, and 721.83 LMH at 0.5, 1, 1.5, and 2 bar, respectively. In contrast, the PAA/PAH-coated UP150 membrane exhibited significantly lower flux values of 93.95, 192.40, 306.46, and 388.76 LMH under the same pressures. This substantial decline corresponds well with the observed 46.7% reduction in MWCO and 13.4% decrease in porosity due to the coating.

When contaminant concentrations were increased, the average flux of the unmodified UP150 membrane under the TMPs of 0.5, 1, 1.5, and 2 bar was slightly affected, yielding 197.26, 387.08, 576.00, and 706.58 LMH. However, for the coated UP150 membrane under the same TMPs, flux values decreased more noticeably to 82.35, 189.12, 292.96, and 369.43 LMH, indicating a higher sensitivity to concentration-related fouling. These findings suggest that while the uncoated membrane’s limited PFAS rejection remains largely unaffected by pollutant load, the enhanced rejection provided by the coating comes at the cost of greater flux decline under higher contaminant levels.

Similarly, the average flux of the UP020 membrane under the TMPs of 1, 2, 3, and 4 bar was 68.89, 144.93, 274.46, and 346.69 LMH, respectively. After coating, flux values under the same TMPs were reduced to 33.68, 74.45, 151.26, and 212.75 LMH. These reductions are consistent with a 40% decrease in MWCO and a 7.2% reduction in porosity. At elevated contaminant concentrations, the uncoated UP020 membrane maintained comparable fluxes (64.64, 144.69, 254.95, and 344.87 LMH), while the coated membrane experienced a further drop to 29.86, 69.80, 133.71, and 189.96 LMH. Again, this trend reflects that increased contaminant rejection due to coating is accompanied by a more pronounced flux decline in response to higher pollutant loads, similar to the behavior observed in the UP150 membrane.

The average flux of the NP030 membrane under the increasing TMPs of 7, 8, 9, and 10 bar was recorded as 48.62, 54.13, 63.48, and 75.65 LMH, respectively. When the contaminant concentration was elevated, a slight decline in flux was observed, yielding average values of 45.60, 49.51, 57.44, and 70.51 LMH under the same TMPs. For the BW30XLE membrane, average fluxes under 9, 11, 13, and 15 bar were determined as 31.28, 35.97, 43.69, and 51.46 LMH, respectively. Upon increasing contaminant concentration, a more pronounced flux reduction was noted, with average values decreasing to 24.73, 32.27, 39.69, and 47.38 LMH. These trends indicate that while both membranes experience a reduction in flux under higher contaminant loads, BW30XLE is more adversely affected, likely due to its tighter pore structure and higher rejection capability. Furthermore, the high flux performance of the PAA/PAH-coated UP020 membrane under low-pressure operation conditions highlights the potential of this modification strategy for membranes with smaller pore sizes.

After treatment with synthetic solutions, upon examining the SEM images provided in the Supplementary Material, no significant changes in the membrane surface characteristics were observed, and it was found that both the coatings and the membrane integrity were maintained. To further investigate the surface-level interaction between PFAS compounds and membrane materials, EDX elemental analysis was performed before and after PFAS exposure. The results, provided in the Supplementary Materials, showed no significant fluorine accumulation on the membrane surfaces, including coated and uncoated samples. This supports the conclusion that PFAS rejection was not driven by adsorption onto the membrane surface but rather by electrostatic repulsion and size exclusion. The absence of fluorine signals complements the FTIR findings and reinforces the hypothesis that the observed removal was primarily due to charge-based mechanisms.

**Figure 7:**
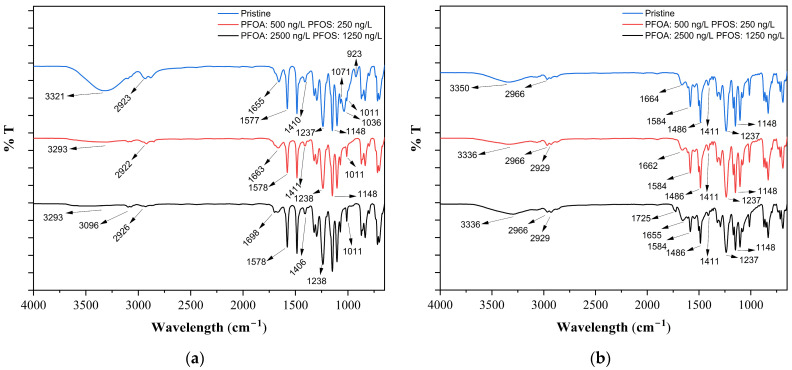

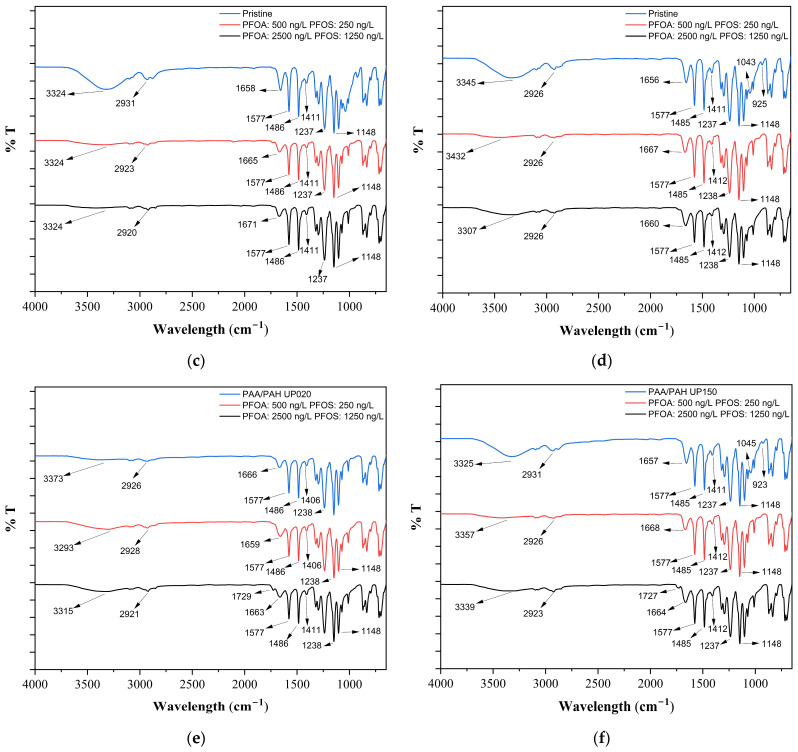
ATR-FTIR spectra: (**a**) NP030 membrane (pristine and PFAS-treated); (**b**) BW30XLE membrane (pristine and PFAS-treated); (**c**) UP020 membrane (pristine and PFAS-treated); (**d**) UP150 membrane (pristine and PFAS-treated); (**e**) PAA/PAH-coated UP020 membrane (before and after PFAS-treatment); (**f**) PAA/PAH-coated UP150 membrane (before and after PFAS-treatment).

ATR-FTIR analyses were conducted to investigate surface-level modifications that may have affected membrane performance. The obtained spectra are shown in Figure 7.

ATR-FTIR analysis revealed a series of spectral modifications that may be associated with changes in the surface chemistry of the membranes after exposure to PFAS compounds. In the case of the UP150 membrane, the characteristic bands at 3325 and 2931 cm^−1^ attributed to –OH/–NH and aliphatic –CH_2_ stretching vibrations appeared largely unchanged following PFAS exposure, while the persistence of PES-specific peaks such as the one at 1043 cm^−1^ suggests limited interaction with the membrane surface. For the PAA/PAH-coated UP150 membrane, the retention of PES-related bands, including those at 923 and 1043 cm^−1^ might indicate that the coating was not sufficiently continuous to fully mask the underlying polymer structure. However, the appearance of a new band at 1727 cm^−1^ following exposure to highly concentrated PFAS solutions may point to some degree of surface interaction involving carboxylic functional groups. The pristine UP020 membrane exhibited characteristic bands at 1658, 1411, and 1237 cm^−1^, typical of PES structures, with relatively minor spectral changes upon PFAS contact, except for the emergence of a weak C=O band around 1671 cm^−1^ at high concentrations, which may suggest weak chemical association. In contrast, the PAA/PAH-coated UP020 membrane displayed a new band at 1729 cm^−1^ might reflect the presence of carboxylic acid functionalities from PFOA and PFOS interacting with the coated surface. The NP030 membrane showed notable spectral shifts as well; the loss of peaks at 1071, 1036, and 923 cm^−1^ and a shift from 1410 to 1406 cm^−1^ suggest possible structural modifications on the membrane surface following PFAS adsorption. Like PAA/PAH-coated UP020 membrane, the BW30XLE membrane displayed a new band at 1725 cm^−1^, which could be attributed to strong interaction between the membrane surface and PFAS carboxylic groups [46,47,48,49,50,51,52,53,54,62]. Overall, the results suggest that significant surface chemical interactions may occur in coated UP020 and BW30XLE membranes, as supported by distinct spectral changes, whereas such effects appear more limited in membranes like UP150 that possess larger pore structures.

## 4. Discussion

This study aimed to enhance the PFAS rejection performance of UF membranes by altering their surface properties through the application of PAA/PAH polyelectrolyte multilayer coatings via deep coating. The hypothesis of the study was that increased surface negativity and reduced pore size through coating would improve the electrostatic repulsion and size-exclusion-based rejection of anionic PFAS compounds such as PFOA and PFOS. The findings strongly support this hypothesis and offer meaningful implications in the context of both membrane science and water treatment applications.

The unmodified UP150 and UP020 membranes exhibited limited rejection capabilities for PFOS and PFOA, primarily due to their large MWCOs and relatively weak surface charges. After coating, significant improvements were observed in removal efficiency, reaching up to 45.3% and 77.8% for PFOS and 43.4% and 73.3% for PFOA, respectively, for UP150 and UP020. These results are consistent with previous studies that highlight the critical role of zeta potential in PFAS removal. For instance, Zhao et al. reported that membranes with more negative zeta potentials exhibited enhanced PFOS rejection via electrostatic repulsion and size exclusion mechanisms [58]. Similarly, Wang et al. observed a direct correlation between increasing PFAS concentrations and improved rejection, likely due to intensified membrane-contaminant interactions [57]. Zeng also reported that membranes with more negative zeta potentials showed higher rejection tendencies for PFHxA, achieving up to 95% removal efficiency [23].

Although the surface-modified UF membranes did not reach the separation levels of dense NF or RO membranes (e.g., BW30XLE achieved >90% removal), the performance of coated UP020 approached that of the NP030 membrane. This aligns with the findings of Tang et al., who indicated that zeta potential effects become less significant in membranes with tighter pore structures [60], whereas surface charge modification in UF membranes can lead to substantial improvements. Furthermore, the results demonstrate that surface modification can significantly narrow the performance gap between UF and NF membranes under low-pressure conditions. These findings are particularly promising given the operational cost advantages of UF membranes compared to high-pressure systems. In addition, Olimattel et al. previously demonstrated that polyelectrolyte-coated UF membranes achieved up to 30% higher PFAS removal compared to unmodified membranes [12], a finding consistent with the 35–40% improvement observed in this study.

The decrease in flux was more pronounced under higher PFAS concentrations, particularly for coated membranes. This trade-off highlights the need for optimization strategies that balance permeability and selectivity.

## 5. Conclusions

This study demonstrated that surface modification of ultrafiltration membranes via PAA/PAH polyelectrolyte multilayer coatings significantly enhances PFAS rejection, particularly for PFOA and PFOS. The application of the deep coating technique reduced MWCO and porosity while increasing the negative surface charge and hydrophilicity of the membranes, leading to improved electrostatic repulsion and size-based exclusion. The key findings are summarized as follows:

- The application of seven-layer PAH/PAA coatings resulted in a substantial increase in negative surface charge, reducing the zeta potential from −16.8 mV to −32.4 mV for UP150 and from −13.2 mV to −30.1 mV for UP020, thereby enhancing electrostatic repulsion against anionic PFAS molecules.
- Despite having larger nominal MWCO values (150 and 20 kDa), the coated membranes achieved remarkable PFAS removal, with PFOA and PFOS rejection rates increasing from ~2–34% to 43–78% depending on membrane type and pollutant concentration.
- Coating led to a measurable decrease in both MWCO (46.7% for UP150, 40% for UP020) and porosity (13.4% and 7.2%, respectively), supporting the presence of a tighter and more selective separation layer.
- Among the tested membranes, coated UP020 achieved PFAS removal efficiencies comparable to those of the NF membrane (NP030), indicating that surface charge engineering can effectively compensate for larger pore sizes in UF membranes.
- FTIR analysis revealed minor spectral changes after PFAS exposure (e.g., appearance of a 1727 cm^−1^ band), suggesting limited surface interaction, while EDX results showed no fluorine accumulation on the membrane surface, indicating that adsorption was not the dominant retention mechanism.

The findings highlight that no significant membrane fouling was observed during short-term experiments, and characterization results confirmed that enhanced electrostatic repulsion driven by increased negative zeta potential was the dominant separation mechanism, with minimal evidence of PFAS adsorption. Overall, this study demonstrates that surface charge engineering through multilayer polyelectrolyte coating is a promising and low-pressure strategy for effective PFAS removal in membrane-based water treatment applications.

### Future Challenges

Although this study demonstrates the promising potential of polyelectrolyte multilayer-coated UF membranes for enhanced PFAS rejection under low-pressure conditions, several key directions remain open for further development. One important area involves the precise control of coating thickness and uniformity during the LbL deposition process. Optimizing the number of bilayers and crosslinking conditions may allow for better membrane permeability and selectivity. Additionally, while short-term performance was successfully demonstrated, evaluating the long-term operational stability, mechanical durability, and anti-fouling properties of the coated membranes under real water matrices—such as municipal wastewater or contaminated groundwater—is crucial for assessing their practical applicability.

Long-term stability and fouling resistance of the coated membranes under real water matrices should also be investigated to assess their practical applicability. Moreover, while this study focused on PFOS and PFOA, two of the most dominant PFAS compounds in the environment, as model compounds, future investigations should extend to short-chain PFAS species, which are known to be more mobile and difficult to remove. Exploring alternative or synergistic polyelectrolyte pairs and advanced surface engineering techniques may further enhance the rejection performance. Collectively, these directions will help bridge the gap between laboratory-scale membrane modification and real-world water treatment applications, thereby maximizing the impact and practicality of this approach. Finally, techno-economic analysis and life-cycle assessment would be valuable to assess the scalability and sustainability of the proposed modification strategy for full-scale water treatment systems.

## Figures and Tables

**Table 1:** Properties of the membranes used in the study.

Membrane	Type	Materials	Molecular Weight Cut Off (MWCO) (kDA)	pH Range
UP150	UF	PES ^1^	150 kDA	1–14
UP020	UF	PES	20 kDA	0–14
NP030	NF	PES	0.5 kDA	0–14
BW30XLE	RO	PA-TFC ^2^	0.1 kDA	1–13

^1^ Polyethersulfone. ^2^ Polyamide thin-film-composite.

## Data Availability

All the datasets presented in this article are not readily available because the data are part of an on-going study.

## Supplementary Materials

The following supporting information can be downloaded at https://www.mdpi.com/article/10.3390/membranes15060172/s1.
