# Supplementary material for: Improving PFAS Rejection by Ultrafiltration Membranes via Polyelectrolyte Multilayer Coating
Source: Membranes (Basel). 2025 Jun 7;15(6):172. doi: 10.3390/membranes15060172 (PMC12195447; doi:10.3390/membranes15060172)
Supplement: Supplementary file 1 [file membranes-15-00172-s001.zip › membranes-3658392-supplementary.pdf]

Figure S1) SEM images of pristine NP030 membrane: left) 10,000× magnification, right) 1,000× magnification

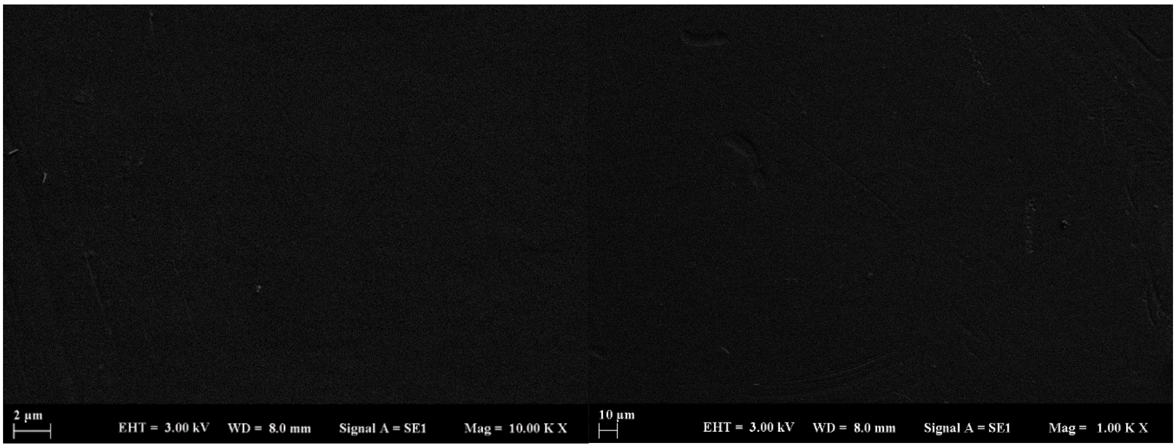

Figure S2) SEM images of NP030 membrane treatment after 500 ng/L PFOS: 250 ng/L : left) 10,000× magnification, right) 1,000× magnification

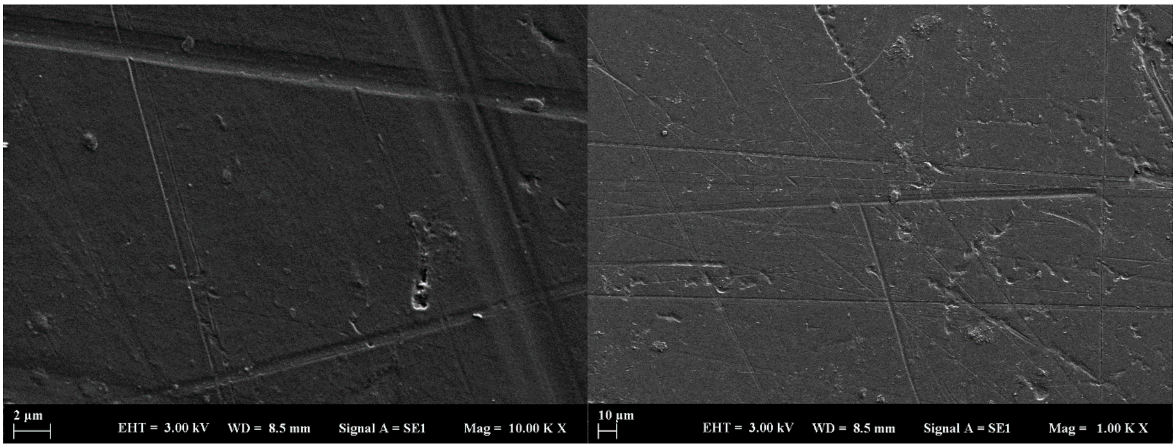

Figure S3) SEM images of NP030 membrane treatment after 2500 ng/L PFOS: 1250 ng/L : left) 10,000× magnification, right) 1,000× magnification

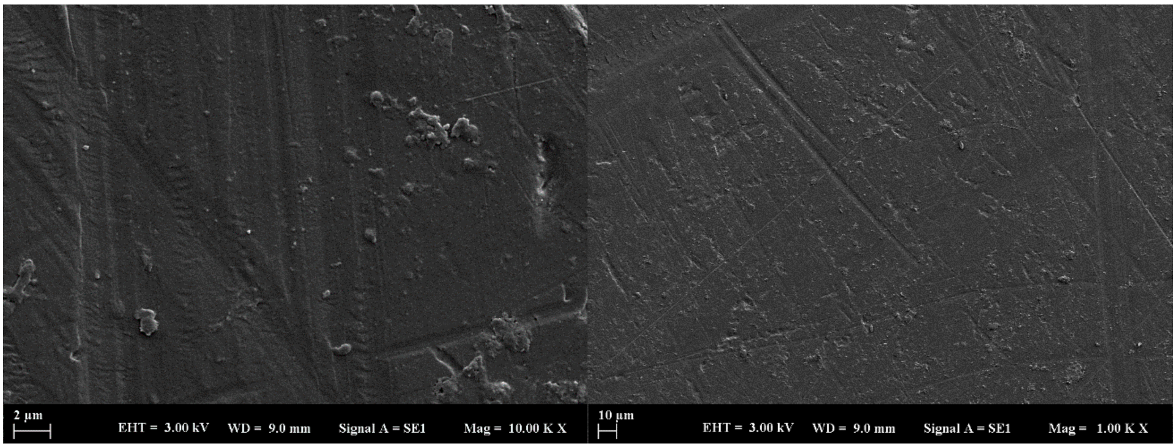

Figure S4) SEM images of pristine BW30XLE membrane: left) 10,000× magnification, right) 1,000× magnification

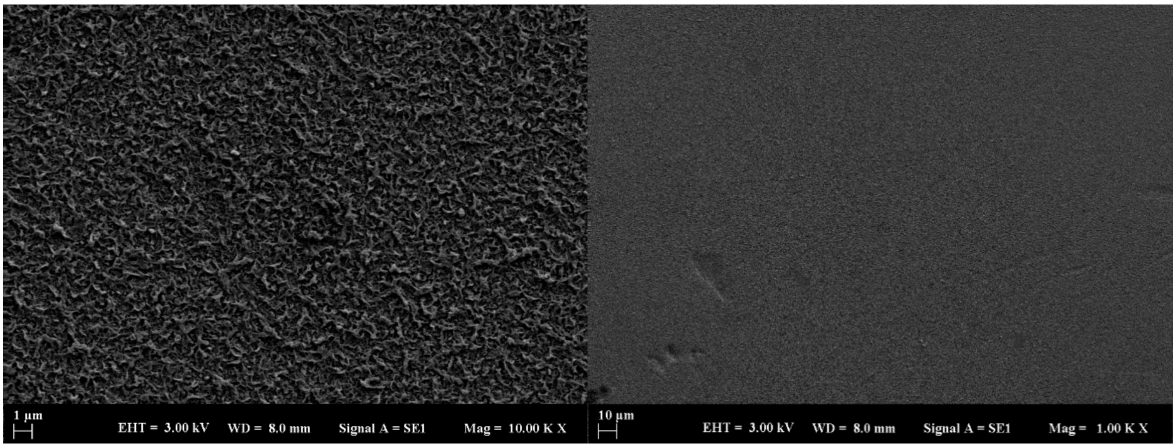

Figure S5) SEM images of BW30XLE membrane treatment after 500 ng/L PFOS: 250 ng/L : left) 10,000× magnification, right) 1,000× magnification

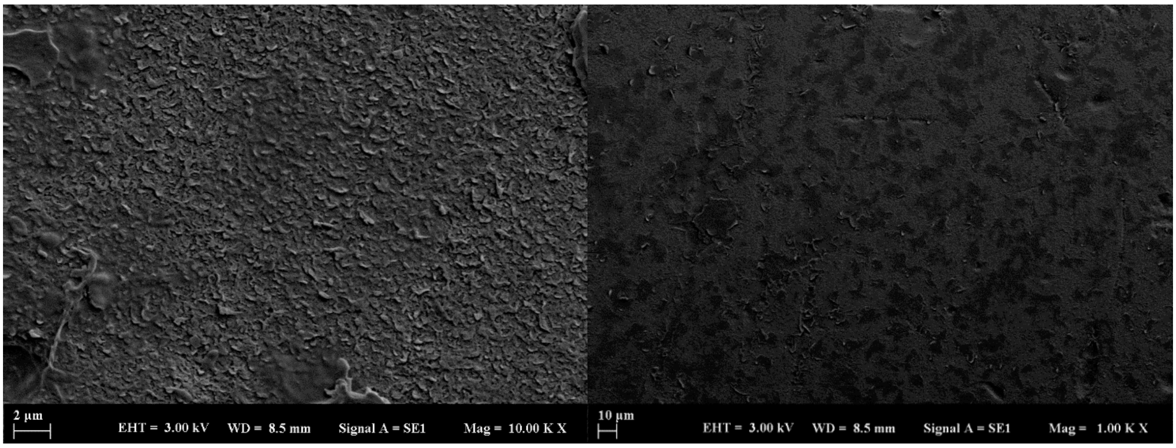

Figure S6) SEM images of BW30XLE membrane treatment after 2500 ng/L PFOS: 1250 ng/L : left) 10,000× magnification, right) 1,000× magnification

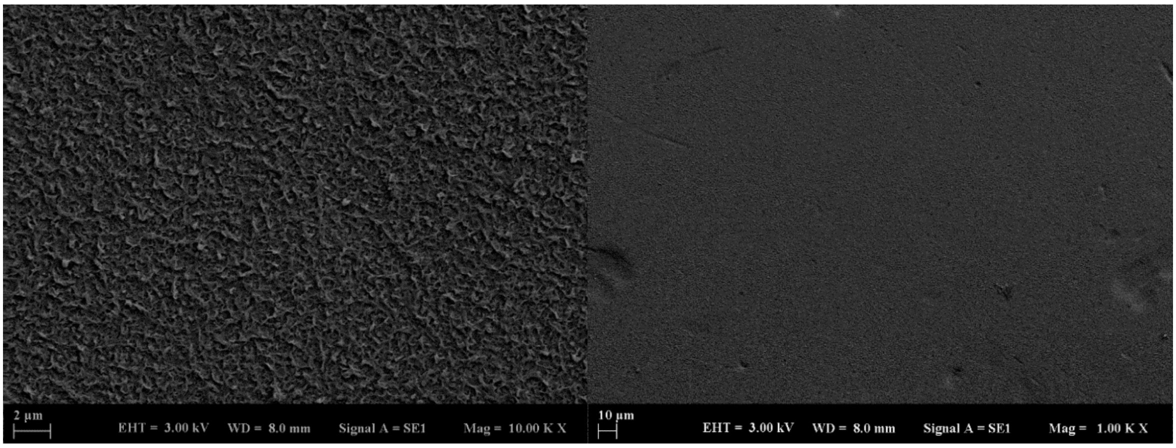

Figure S7) SEM images of pristine UP020 membrane: left) 10,000× magnification, right) 1,000× magnification

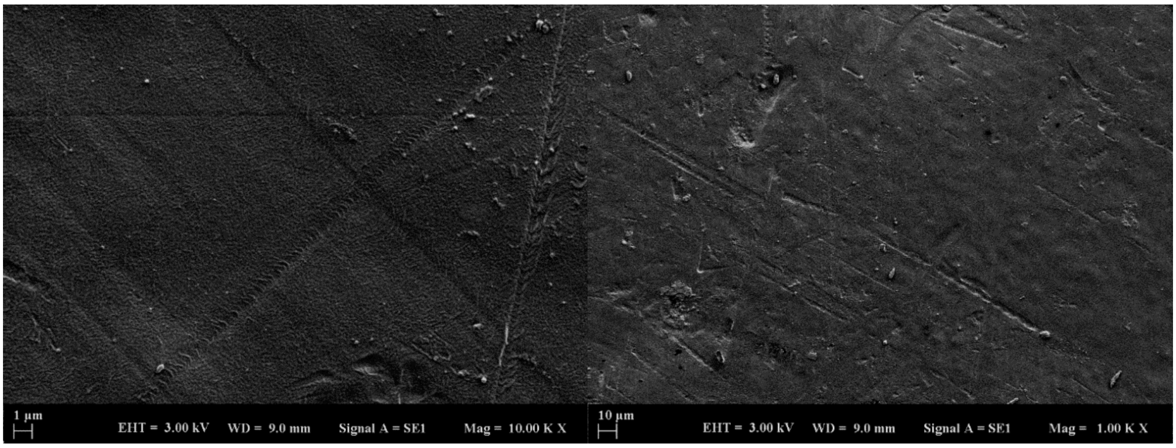

Figure S8) SEM images of UP020 membrane treatment after 500 ng/L PFOS: 250 ng/L : left) 10,000× magnification, right) 1,000× magnification

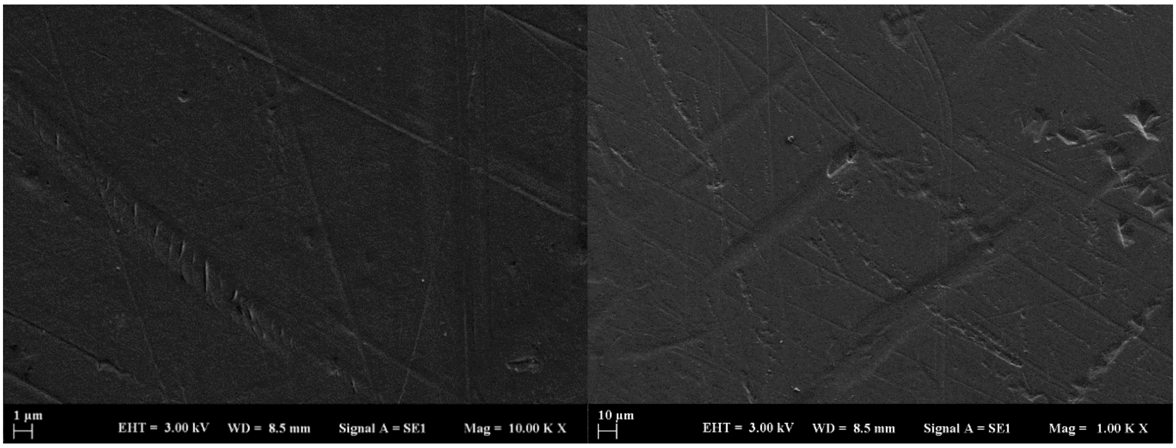

Figure S9) SEM images of UP020 membrane treatment after 2500 ng/L PFOS: 1250 ng/L : left) 10,000× magnification, right) 1,000× magnification

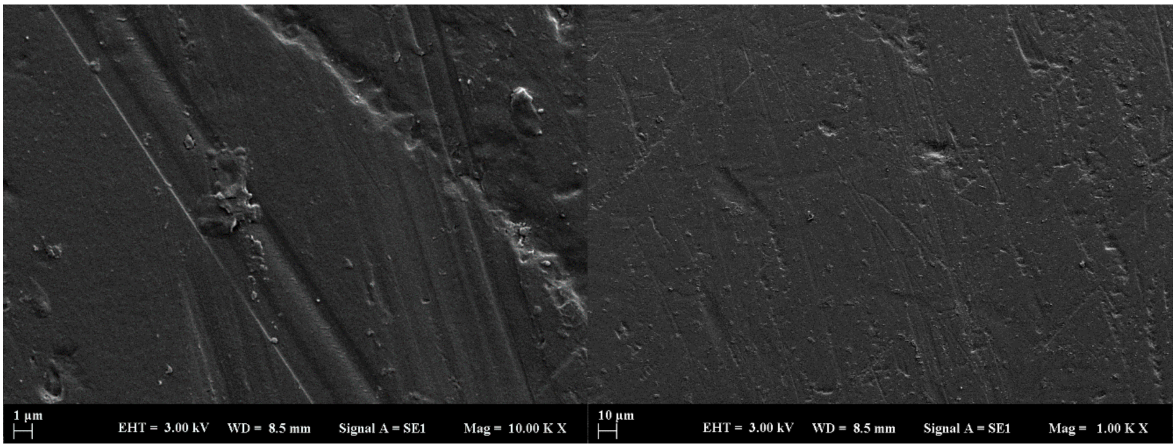

Figure S10) SEM images of pristine UP150 membrane: left) 10,000× magnification, right) 1,000× magnification

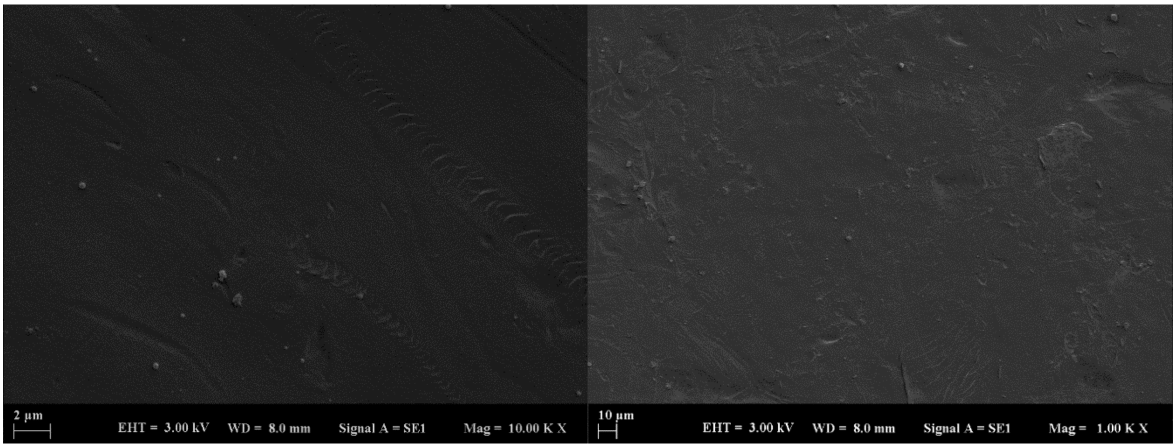

Figure S11) SEM images of UP150 membrane treatment after 500 ng/L PFOS: 250 ng/L : left) 10,000× magnification, right) 1,000× magnification

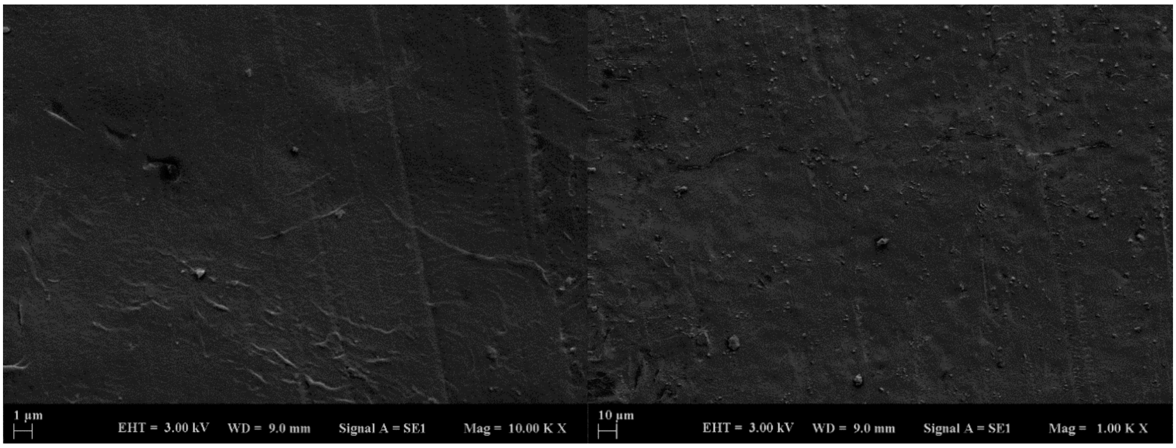

Figure S12) SEM images of UP150 membrane treatment after 2500 ng/L PFOS: 1250 ng/L : left) 10,000× magnification, right) 1,000× magnification

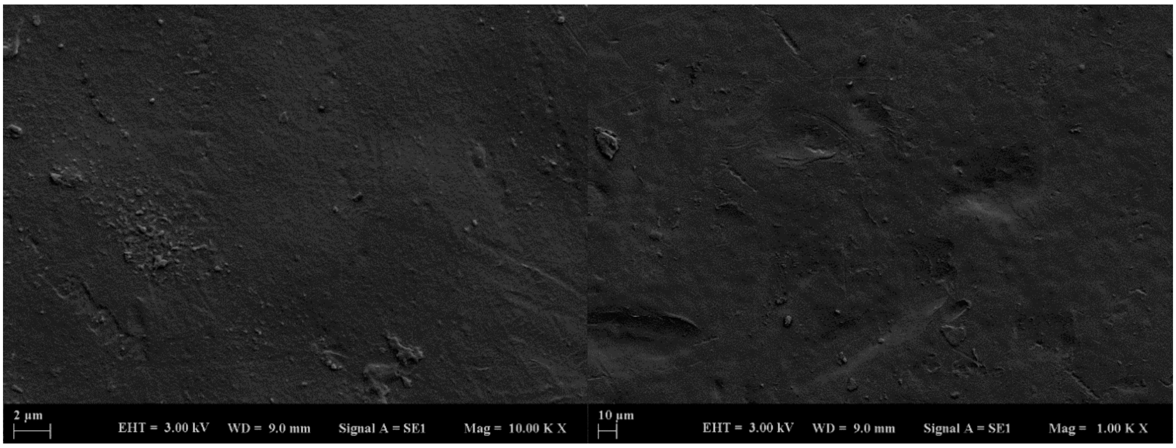

Figure S13) SEM images of PAA/PAH coated UP020 membrane: left) 10,000× magnification, right) 1,000× magnification

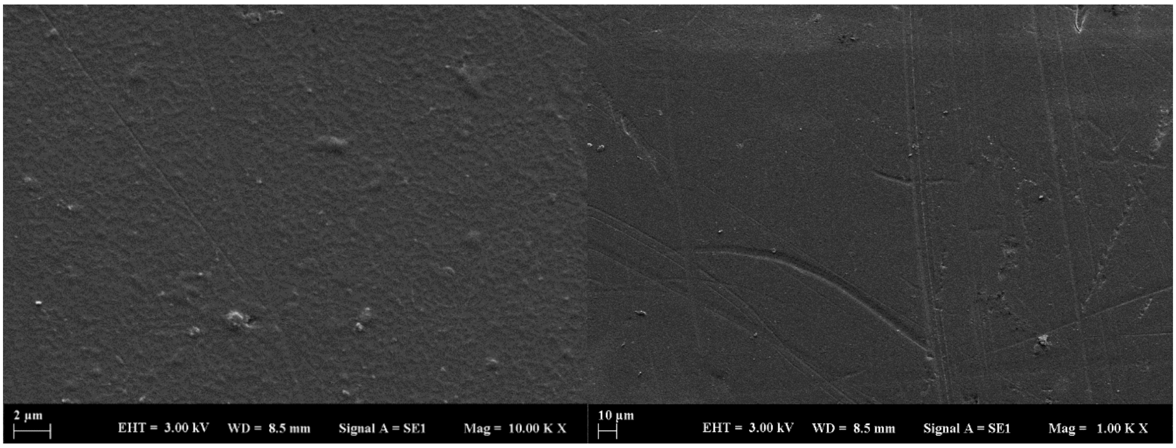

Figure S14) SEM images of of PAA/PAH coated UP020 membrane treatment after 500 ng/L PFOS: 250 ng/L : left) 10,000× magnification, right) 1,000× magnification

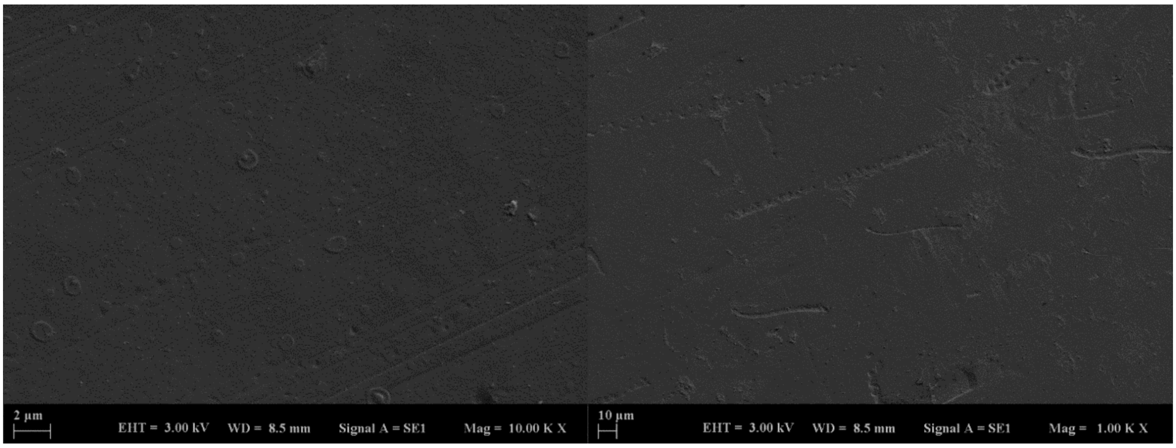

Figure S15) SEM images of of PAA/PAH coated UP020 membrane treatment after 500 ng/L PFOS: 250 ng/L : left) 10,000× magnification, right) 1,000× magnification

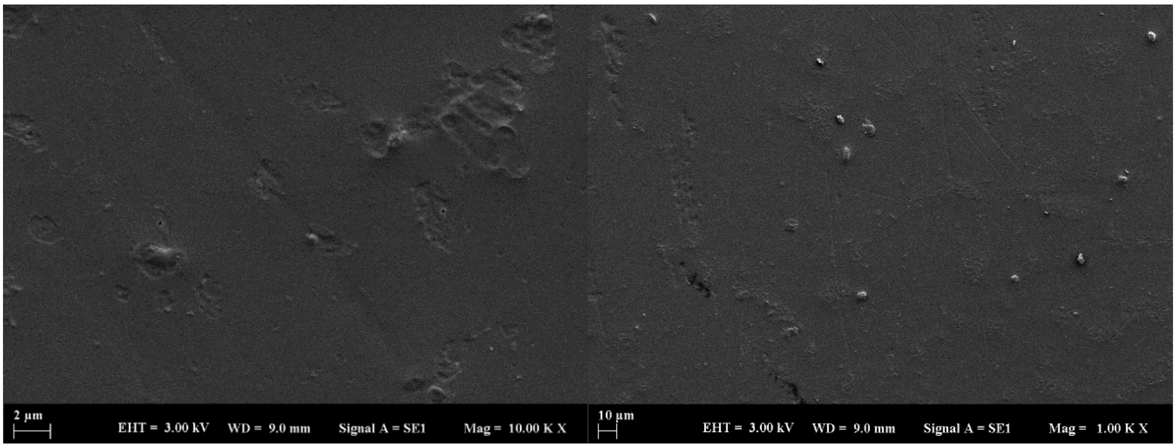

Figure S16) SEM images of PAA/PAH coated UP150 membrane: left) 10,000× magnification, right) 1,000× magnification

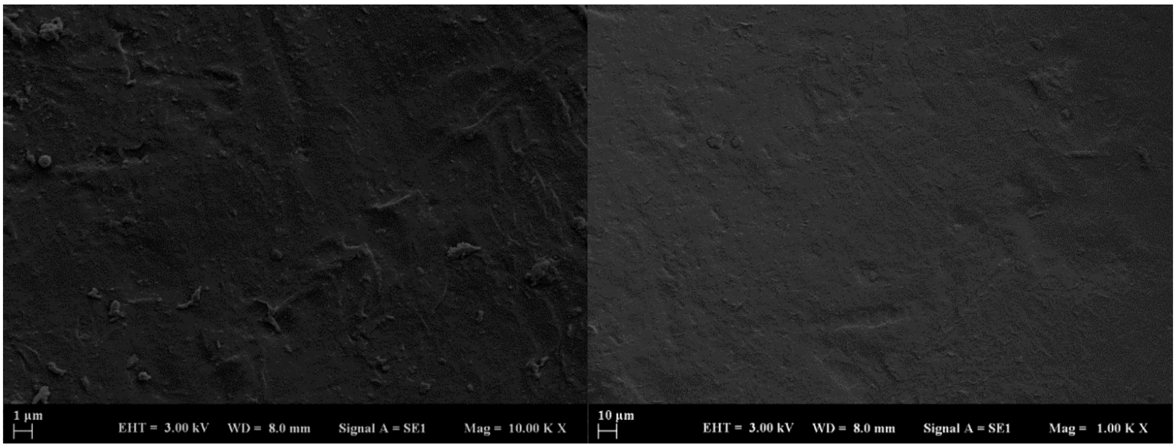

Figure S17) SEM images of of PAA/PAH coated UP150 membrane treatment after 500 ng/L PFOS: 250 ng/L : left) 10,000× magnification, right) 1,000× magnification

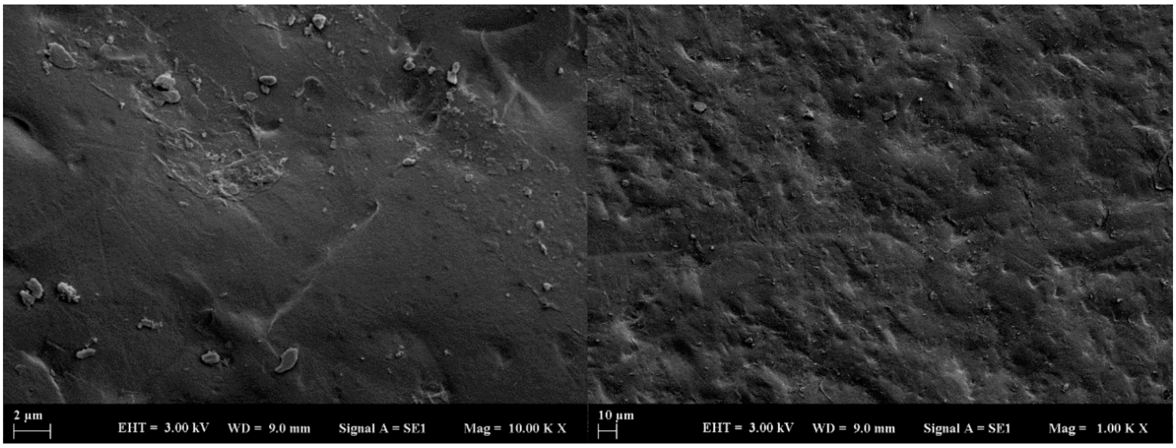

Figure S18) SEM images of of PAA/PAH coated UP150 membrane treatment after 2500 ng/L PFOS: 1250 ng/L : left) 10,000× magnification, right) 1,000× magnification

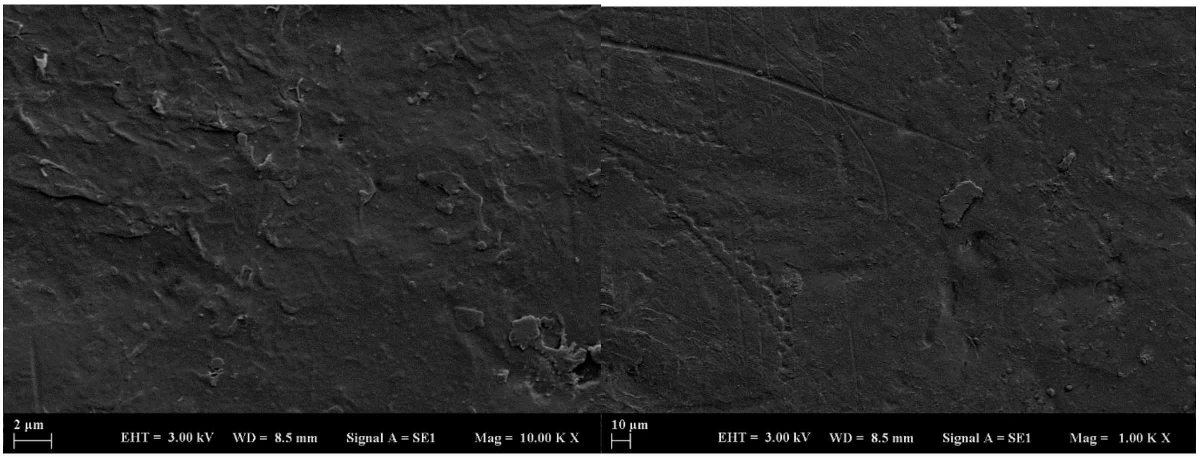

Element	Weight %	Atomic %
**C K**	56.74	68.30
**N K**	0.00	0.00
**O K**	26.92	24.33
**F K**	0.00	0.00
**S K**	16.34	7.37

Table S1) Elemental composition of pristine NP030 membrane

Element	Weight %	Atomic %
**C K**	58.02	70.63
**N K**	0.00	0.00
**O K**	22.33	20.40
**F K**	0.00	0.00
**S K**	19.65	8.96

Table S2) Elemental composition of NP030 membrane treatment after 500 ng/L PFOS: 250 ng/L

Element	Weight %	Atomic %
**C K**	58.44	71.32
**N K**	0.00	0.00
**O K**	21.09	19.32
**F K**	0.00	0.00
**S K**	20.47	9.36

Table S3) Elemental composition of NP030 membrane treatment after 2500 ng/L PFOS: 1250 ng/L

Element	Weight %	Atomic %
**C K**	71.69	80.40
**N K**	1.99	1.92
**O K**	15.72	13.24
**F K**	0.00	0.00
**S K**	10.59	4.45

Table S4) Elemental composition of pristine BW30XLE membrane

Element	Weight %	Atomic %
**C K**	74.21	83.05
**N K**	0.69	0.66
**O K**	13.68	11.50
**F K**	0.00	0.00
**S K**	11.42	4.79

Table S5) Elemental composition of BW30XLE membrane treatment after 500 ng/L PFOS: 250 ng/L

Element	Weight %	Atomic %
**C K**	72.76	81.37
**N K**	1.46	1.40
**O K**	15.19	12.75
**F K**	0.16	0.11
**S K**	10.44	4.37

Table S6) Elemental composition of BW30XLE membrane treatment after 2500 ng/L PFOS: 1250 ng/L

Element	Weight %	Atomic %
**C K**	52.54	63.48
**N K**	0.00	0.00
**O K**	33.09	30.01
**F K**	0.00	0.00
**S K**	14.35	6.49

Table S7) Elemental composition of pristine UP020 membrane:

Element	Weight %	Atomic %
**C K**	57.97	71.32
**N K**	0.00	0.00
**O K**	20.09	18.56
**F K**	0.00	0.00
**S K**	21.94	10.11

Table S8) Elemental composition of UP020 membrane treatment after 500 ng/L PFOS: 250 ng/L

Element	Weight %	Atomic %
**C K**	58.35	71.52
**N K**	0.00	0.00
**O K**	20.29	18.67
**F K**	0.00	0.00
**S K**	21.36	9.81

Table S9) Elemental composition of UP020 membrane treatment after 2500 ng/L PFOS: 1250 ng/L

Element	Weight %	Atomic %
**C K**	59.86	71.14
**N K**	0.00	0.00
**O K**	24.58	21.93
**F K**	0.00	0.00
**S K**	15.56	6.93

Table S10) Elemental composition of pristine UP150 membrane treatment

Element	Weight %	Atomic %
**C K**	59.21	71.09
**N K**	0.36	0.38
**O K**	22.71	20.47
**F K**	0.00	0.00
**S K**	17.41	7.83

Table S11) Elemental composition of UP150 membrane treatment after 500 ng/L PFOS: 250 ng/L

Element	Weight %	Atomic %
**C K**	58.16	70.54
**N K**	0.00	0.00
**O K**	22.92	20.87
**F K**	0.00	0.00
**S K**	18.91	8.59

Table S12) Elemental composition of UP150 membrane treatment after 2500 ng/L PFOS: 1250 ng/L

Element	Weight %	Atomic %
**C K**	59.02	72.12
**N K**	0.00	0.00
**O K**	19.86	18.22
**F K**	0.00	0.00
**S K**	21.11	9.66

Table S13) Elemental composition of PAA/PAH coated UP020 membrane

Element	Weight %	Atomic %
**C K**	58.50	71.59
**N K**	0.00	0.00
**O K**	20.38	18.72
**F K**	0.00	0.00
**S K**	21.12	9.68

Table S14) Elemental composition of PAA/PAH coated UP020 membrane treatment after 500 ng/L PFOS: 250 ng/L

Element	Weight %	Atomic %
**C K**	60.37	73.07
**N K**	0.00	0.00
**O K**	19.68	17.88
**F K**	0.00	0.00
**S K**	19.95	9.04

Table S15) Elemental composition of PAA/PAH coated UP020 membrane treatment after 2500 ng/L PFOS: 1250 ng/L

Element	Weight %	Atomic %
**C K**	52.79	63.74
**N K**	2.94	3.05
**O K**	28.94	26.23
**F K**	0.00	0.00
**S K**	15.19	6.87

Table S16) Elemental composition of PAA/PAH coated UP150 membrane

Element	Weight %	Atomic %
**C K**	59.88	72.21
**N K**	0.03	0.03
**O K**	21.29	19.27
**F K**	0.00	0.00
**S K**	18.80	8.49

Table S17) Elemental composition of PAA/PAH coated UP150 membrane treatment after 500 ng/L PFOS: 250 ng/L

Element	Weight %	Atomic %
**C K**	59.85	72.49
**N K**	0.04	0.04
**O K**	20.34	18.50
**F K**	0.00	0.00
**S K**	19.76	8.97

Table S18) Elemental composition of PAA/PAH coated UP150 membrane treatment after 2500 ng/L PFOS: 1250 ng/L
